# Effectiveness of a Brief Dietetic Intervention for Hyperlipidaemic Adults Using Individually-Tailored Dietary Feedback

**DOI:** 10.3390/healthcare4040075

**Published:** 2016-10-11

**Authors:** Tracy L. Schumacher, Tracy L. Burrows, Megan E. Rollo, Neil J. Spratt, Robin Callister, Clare E. Collins

**Affiliations:** 1 School of Health Sciences, Faculty of Health and Medicine, University of Newcastle, Callaghan 2308, NSW, Australia; tracy.schumacher@newcastle.edu.au (T.L.S.); tracy.burrows@newcastle.edu.au (T.L.B.); megan.rollo@newcastle.edu.au (M.E.R.); 2 Priority Research Centre for Physical Activity and Nutrition, University of Newcastle, Callaghan 2308, NSW, Australia; robin.callister@newcastle.edu.au; 3 Priority Research Centre for Translational Neuroscience and Mental Health & Faculty of Health and Medicine, University of Newcastle, Callaghan 2308, NSW, Australia; neil.spratt@newcastle.edu.au; 4 Hunter New England Local Health District & Hunter Medical Research Institute, New Lambton Heights 2305, NSW, Australia; 5 School of Biomedical Sciences and Pharmacy, University of Newcastle, Callaghan 2308, NSW, Australia

**Keywords:** cardiovascular disease, hyperlipidaemia, diet, dietitian, food patterns, nutrition, counselling

## Abstract

Dietary modifications can improve serum lipids and reduce cardiovascular disease (CVD) risk. However, attendance at multiple dietary consultations can be a barrier to achieving behaviour change. This study investigated the effectiveness of a brief dietetic intervention on CVD risk factors in hyperlipidaemic adults. Adults with total cholesterol ≥ 5.0 mmol/L or low density lipoprotein (LDL) cholesterol ≥ 4.0 mmol/L and not currently taking lipid-lowering medication were eligible for a minimum 6-week dietary intervention. Dietary intake data and blood lipids were acquired prior to a single counselling session with an Accredited Practising Dietitian (APD). The intervention used targeted feedback with purpose-developed education materials to supplement advice. CVD risk factors and dietary intakes were used to assess pre-post intervention change using linear mixed model regression analyses. Thirty-nine participants (59.3 ± 11.1 years, n = 28 female) were analysed. Mean ± SD follow-up from baseline time was 9.5 ± 2.5 weeks. Significant (*p* < 0.05) reductions in total cholesterol (−0.51 mmol/L), total:HDL (high density lipoprotein) ratio (−0.27 mmol/L), triglycerides (−0.38 mmol/L), total energy (−870 kJ/day), energy from nutrient-poor foods (−1006 kJ/day) and sodium (−325 mg/day), and improved dietary fat quality (−5.1% of energy/day saturated, +5.0% of energy/day polyunsaturated) and body mass index (−0.4 kg/m2) were achieved. A brief intervention by an APD incorporating targeted, personalised dietary feedback and education in a single counselling session can improve lipid profiles in adults with hyperlipidaemia.

## 1. Introduction

The World Health Organization identified cardiovascular disease (CVD) as the leading worldwide cause of mortality (46.2%) in 2012. Dietary intakes are a modifiable risk factor for CVD [1,2] with dietary patterns, such as the Mediterranean diet shown to reduce incidence of cardiovascular events within five years of adhering to the pattern [3]. The United States Preventive Services Task Force deemed the likelihood of harm to be small to none for dietary modification, with adequate evidence of benefits from intensive counselling in populations with multiple risk factors for CVD [2].

However, achieving dietary change in populations at increased risk of CVD can be difficult [4,5]. Barriers to implementation of dietetic counselling include the time and cost of multiple dietetic consultations for those at risk, as well as reluctance of medical practitioners to refer at risk patients if the practitioner believes the patient is unwilling or unable to attend dietetic consultations or that medication is going to be as effective [6]. Evidence for the effectiveness of less intensive dietary interventions, i.e., fewer consultations, is lacking and trials assessing such approaches are needed [2]. Given these barriers and lack of evidence for the effectiveness of brief interventions, a study design more pragmatic than explanatory may be of value. Pragmatic study designs mimic or are suitable for normal practice, use participants that would typically be receiving the treatment, and target outcomes relevant to the stakeholders: i.e., the patients, general practitioners and healthcare providers [7].

Key components of behavioural interventions shown to contribute to effectiveness include individualised assessment, goal setting, education and feedback, and provision of written information to reference as verbal information is commonly forgotten or incorrectly recalled [8,9,10]. Also, provision of food items or subsidising healthier food products have been shown to be effective in weight-loss interventions and in modifying dietary behaviours [11,12].

The aim of the current study was to trial the effectiveness of a brief dietetic intervention on diet-related CVD risk factors in hyperlipidaemic adults. The intervention translated the best-available dietary evidence into a manual for participants and included targeted feedback and individualised CVD-health counselling strategies in a single session provided by an Accredited Practising Dietitian (APD). It was hypothesised that the dietetic intervention would improve lipid profiles.

## 2. Materials and Methods

### 2.1. Study Design

**Figure 1:**
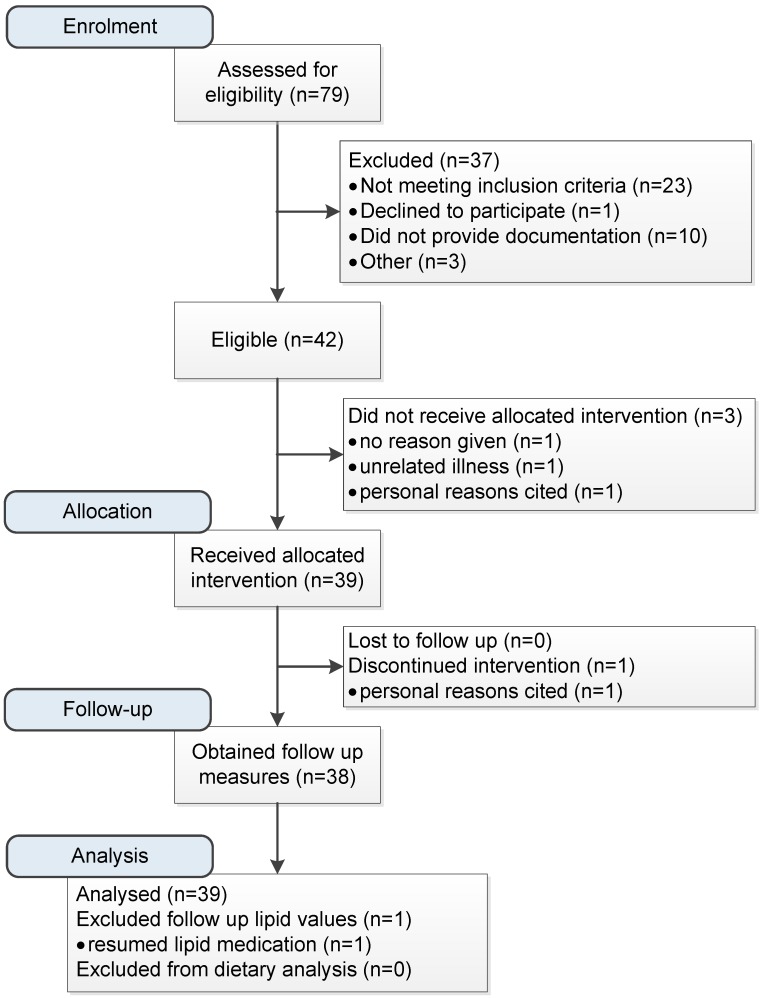
Recruitment, allocation and analysis flowchart.

The study was a pre-post dietary intervention in hyperlipidaemic adults. The study protocol is summarised in Figure 1. Participants completed the Australian Eating Survey (AES) food frequency questionnaire and provided a fasting blood sample for analysis prior to attending a single dietetic counselling session where they received personalised feedback on their current diet, CVD risk, and counselling on dietary strategies. The dietary advice focused on evidence from the Mediterranean and Portfolio diets, which have been shown to be effective in lowering CVD events and risk factors [3,13]. A study-specific education manual was provided to assist participants adopt the recommended eating patterns. Assessments of diet (24-hour recalls) and CVD risk (blood lipids, blood pressure, anthropometric measures) were conducted prior to counselling and after a minimum 6 weeks follow-up, at the participant’s convenience (see Supplementary Figure S1: study timeline). Primary outcome measures were dietary changes and plasma total cholesterol.

### 2.2. Participants

Individuals were eligible to participate if they were aged between 18–75 years, had internet access, were not on lipid lowering medication and had one or more of the following: low density lipoprotein (LDL) cholesterol ≥ 4.00 mmol/L; total:HDL (high density lipoprotein) ratio ≥ 5; total cholesterol ≥ 5.00 mmol/L [14]. Participants currently on medication for hyperlipidaemia were eligible if their treating medical practitioner provided written clearance to halt medication for the duration of the study, and participants undertook a six-week washout period. Those with medical conditions affecting dietary intake (such as coeliac disease) or requiring medication for thyroid conditions were excluded. Written consent was obtained from all participants. Ethics approval was obtained from the University of Newcastle Human Research Ethics Committee (H-2013-0420). Participants were recruited via media releases from the University of Newcastle and the Hunter Medical Research Institute (HMRI), advertising on notice boards within the university setting, invitations through the HMRI volunteer research register, and by word of mouth.

### 2.3. Dietary Assessments

Online questionnaires were used to obtain demographic data, health characteristics, usual dietary intake and preferences for food products related to CVD health. These were completed by participants at their convenience, prior to the dietary intervention. To inform dietary feedback and counselling, usual dietary intakes over the last 6 months were derived from the Australian Eating Survey (AES) [15,16]. The AES is a validated 120 item semi-quantitative food frequency questionnaire (FFQ) that assesses frequency of usual intake over the past six months [15,16]. AES nutrient intakes were computed using FoodWorks version 4.00.1158 (FoodWorks, Xyris Software (Australia) Pty Ltd., Ashwood, Australia, 2005), the Australian AusNut 1999 nutrient database (All Foods, Revision 17) and AusFoods (Brands, Revision 5).

To determine intervention effects on diet, dietary intake was assessed using a three-pass 24-hour recall on two occasions (one weekend day; one week day) at both baseline and follow-up, and was performed by one researcher (Tracy Schumacher) [17,18,19]. Prior to baseline dietary assessment, each participant was issued with a booklet of two dimensional food models that included a reference scale to assist with portion size estimation. Entries obtained from 24-hour recalls were matched to a food from the AUSNUT 2011–2013 food nutrient database by one research dietitian and assessed for comparability by another. Foods not found in the database were matched to foods of similar nutrient content. Quantities were converted to gram measures using values from the AUSNUT food measures database where necessary [20,21]. Energy-dense, nutrient-poor foods, or discretionary food items, were defined according to the Australian Dietary Guidelines and individually categorised using the Australian Health Survey discretionary food list [22]. Examples of discretionary foods include most sweetened biscuits, foods high in saturated fat, salt or sugar. Nutrient intake values for 24-hour recalls were calculated using Stata/IC 13.1 by multiplying gram measures by nutrient values for each individual food [23]. Timing of dietary assessments can be seen in Supplementary Figure S1: Study timeline.

### 2.4. Clinical and Anthropometric Assessments

All clinical and anthropometric measures were collected at the time of the face-to-face intervention. Blood samples collected after an overnight fast were assayed for blood lipid concentrations, glucose, insulin and inflammatory markers at an accredited pathology service laboratory (National Association of Testing Authorities, Newcastle, Australia) at baseline and follow-up. Insulin was measured and HOMA IR calculated only at baseline, unless a result ≥ 10 mIU/L was found. Brachial and central blood pressure and arterial stiffness measures (augmentation index) were obtained with the Pulsecor Cardioscope II (Pulsecor Ltd., Auckland, New Zealand) using WelchAllyn FlexiPort reusable blood pressure cuffs. Participants were seated for five minutes before the first measurement occurred and repeat measures were taken at two-minute intervals until two consistent measures were obtained. Participants’ height and weight were measured in light clothing to 0.1 cm and 0.1 kg, respectively using the Biospace BSM370 Automatic BMI Scale Stadiometer (Biospace Co. Ltd., Seoul, Korea) and used to calculate body mass index (BMI, kg/m^2^). Waist circumference was measured at the narrowest point between the lower costal (10th rib) border and the top of the iliac crest using a non-extensible steel tape (KDFS10-02, KDS Corporation, Osaka, Japan). Physical activity was assessed using the International Physical Activity Questionnaire (IPAQ) long form for the previous seven days [24,25].

### 2.5. Intervention

The intervention was based on a Protection Motivation Theory (PMT) framework, which describes health behaviours as responses to perceptions of threats of vulnerability and their severity [26]. The theory posits that individuals determine the benefits and/or usefulness of performing adaptive (helpful) or mal-adaptive (harmful) behaviours, then assess their confidence to perform the given action [26]. The study sought to use awareness of personal CVD risk as motivation and integration of behaviour change strategies to build self-efficacy for helpful dietary intake tasks, such as increasing fish and reducing energy-dense, nutrient-poor foods, and to provide meaningful feedback on performing the recommended strategies.

Following baseline assessments, written feedback was provided in the form of a booklet, including the individual’s serum cholesterol results, anthropometric measures, and analysis of their usual dietary intake. Dietary feedback included macronutrient and micronutrient intakes, and percentage of energy contributed by core (nutrient dense) and energy-dense, nutrient-poor foods. Verbal feedback was provided in regard to CVD risk factors identified, such as serum lipids, and was linked to dietary intake. Dietetic counselling immediately followed in a 45-minute session, which used a semi-structured presentation to ensure consistent delivery of the dietary components and goal setting strategies, whilst allowing for individualised counselling by the APD (see Table 1). Participants were educated about a range of dietary components that had been demonstrated to reduce CVD risk and informed about heart healthy eating guidelines [3,13,27,28]. Participants were free to choose goals from among the dietary components presented (see Table 1). The Mediterranean diet is characterised by a high monounsaturated:saturated fat ratio, high intakes of legumes, fruits, vegetables, wholegrains, minimally processed cereals, regular intakes of fish and nuts, low intakes of red meats, and moderate intakes of dairy and alcohol [3,29,30]. The Portfolio diet is a predominantly vegetarian diet with the addition of plant-based foods with lipid-lowering properties such as plant sterols, soy and vegetable protein, nuts and soluble fibres [13]. Additional resources included a nutrition education manual specifically designed to complement and extend the dietary information delivered as part of the intervention and included supplementary detail, food guides and recipes for targeted foods. Participants also received a grocery bag containing samples of shelf-stable recommended food products valued at approximately $50 AUD. This was to encourage participants to try foods that were not part of their usual diet, such as soy milk, low-sodium canned beans/legumes and canned oily fish. In the week following the intervention, participants received one follow-up phone call (approximately 10 minutes) from the APD to discuss progress and to troubleshoot any difficulties faced.

Participants were provided with feedback on their dietary intake and physical changes, such as blood pressure and weight at the face-to-face follow-up assessment session. Individual results of the post-intervention blood biomarker analyses were provided to participants via telephone when available.

### 2.6. Statistics

A sample size of 33 participants was required to detect a 0.4 mmol/L change in the primary outcome measure of total cholesterol, based on a standard deviation of 0.8, power of 80% and an alpha of 0.05 [33]. Allowing for 20% loss to follow-up, the study aimed to recruit 40 participants. Data were analysed as intention-to-treat by mixed model linear regression using Stata/IC 13.1 [23]. Analyses for total cholesterol, HDL and total:HDL ratio were adjusted for sex, BMI and physical activity. Analyses for triglycerides, LDL cholesterol, inflammation markers, glucose, blood pressure, and augmentation index were adjusted for sex and BMI. Analyses for dietary measures were adjusted for recall on weekend/weekday and sex, with percentage energy values adjusted for weekend/weekday recall only. Alcohol was tested in lipid analyses where it may have had an effect, and potentially removed in a backward stepwise approach, if inclusion in the model did not improve the data fit and removal did not change the coefficients. Categorical data were analysed by using multinomial logistic regression, with standard errors adjusted for clustering to account for multiple time points.

## 3. Results

Participant flow through the trial is summarised in Figure 1, with 42 participants eligible and 39 participating in the intervention from February to December 2014. Time between baseline and follow-up was (mean ± SD) 9.5 ± 2.5 weeks. Participants were aged 59.3 ± 11.1 years (range 25–73), 72% female, 28, had completed education to higher than Year 10 or equivalent, and 18 had household incomes below $1000 AUD per week. Six participants discontinued their lipid-lowering medication prior to starting the trial, with one restarting after baseline results (follow-up lipid results excluded from analysis). Thirty-four participants reported one or more health conditions: high cholesterol (n = 27), high blood pressure (n = 13), arthritis (n = 10), depression (n = 4), asthma (n = 3), type 1 and 2 diabetes (n = 1 each). No participants were current smokers, but 20 were former smokers. Physical activity levels were categorised by IPAQ as low (n = 6), moderate (n = 21) or high (n = 12) at baseline and low (n = 4), moderate (n = 24) or high (n = 10) at follow-up.

Table 2 summarises baseline, follow-up, and changes in CVD health indicator measures. Significant post-intervention reductions in triglycerides, LDL and total cholesterol, total:HDL cholesterol ratio were observed, including a small reduction in HDL cholesterol. For serum lipids, total cholesterol decreased by 7%, LDL-C by 6% and HDL by 3%, while the ratio of HDL:total cholesterol decreased by 5% and triglycerides by 24%. Significant reductions in BMI, waist circumference and brachial blood pressure were also observed. No change was seen in inflammatory markers (high sensitivity C-reactive protein) or liver function (ALT, AST, GGT).

Table 3 summarises dietary intake measured by 24-hour recalls, indicating a substantial reduction in the proportion of energy from energy-dense, nutrient-poor foods. Macronutrient contributions remained relatively stable, although small but significant changes were found in protein, saturated and polyunsaturated fat, and sodium intakes. Fruit and vegetable intakes remained sub-optimal relative to national recommendations.

Table 4 indicates that some dietary habits related to CVD health, such as using reduced fat cheese, were amendable to change (*p* < 0.01).

## 4. Discussion

Significant reductions in CVD risk factors were achieved through dietary changes implemented by hyperlipidaemic adults following a brief dietetic intervention by an APD based on individualised feedback and advice. Key intervention components were prior assessment of dietary intake using a purpose-designed online questionnaire and access to current blood lipid results so that the single counselling session could be personally targeted, with a focus on providing feedback and education, with support via purposed designed resources. Results suggest that a brief dietetic intervention, suitable for widespread use in primary care settings, can have a valuable impact on diet-related CVD risk factors.

Participants improved overall serum lipid profiles, which can be attributed to dietary change, given that other confounding variables, such as physical activity, medication and smoking status were accounted for in the statistical analysis. Findings from the Cholesterol Treatment Trialists’ Collaboration indicated that a 1.00 mmol/L reduction in serum LDL cholesterol following statin prescription reduced the major vascular events rate ratio by 0.62, 0.69 and 0.79 in those at < 5%, ≥ 5 to < 10% and ≥ 10 to < 20% five-year risk, respectively [37]. An additional benefit of achieving reductions in LDL cholesterol secondary to dietary change is the concomitant reduction in risk for other chronic conditions [38]. Reductions in LDL (6%) and total cholesterol (7%) in the current study are similar to those achieved using a single-session community-based intervention by Gaetke et al., where participants were free to choose their own food based on advice provided [39], but were less than the 26% and 34% reductions reported by Tovar et al. in an intervention that specified menu plans and provided prescribed foods [40].

The dietary change responsible for the greatest reduction in energy intake in the current study was the reduction in energy-dense, nutrient-poor food consumption from 32% of total energy at baseline to 24% at follow up. Increased intakes of core foods were small and individually variable. There were no group differences in intakes of recommended foods, such as fruit and vegetables, and only small increases in legumes and soy. The significant increases in soy and plant sterol containing foods indicate that many were willing to increase intakes of these foods, but further strategies are needed to reach the recommended efficacious levels [41,42,43]. There were no significant group level improvements in reported intakes of nuts, fish, fruit, fibre from oats/psyllium/linseed or vegetables. However, with the exception of vegetables, for which legumes may have been substituted, the point estimates were in the direction of improvement. Individual participants made changes in some but not all of the food recommendations, and this varied based on personal preferences and baseline consumption. The current study provides evidence for the cumulative benefit of multiple small improvements in intakes of foods with known cardio-protective effects being sufficient to provide CVD-risk protection, even though individual food changes were not detected as statistically significant.

The PREDIMED study was a large scale intervention that provided evidence of the long-term impact of a Mediterranean style eating pattern on the primary prevention of CVD [3]. Total fat intakes (33% energy) of those in the current study were substantially less than those in the PREDIMED study (41% post-trial), although they were still higher than Australian adults at 31% [3,44]. Consequently there were slightly higher protein (3%) and carbohydrate (3%) intakes compared to PREDIMED. Total fibre intakes in the current study were adequate, although the types of foods consumed were unlikely to contain the level or viscosity of soluble fibres required to reduce risk factors for CVD [45,46]. Future quantification of soluble and insoluble fibre intakes could be a valuable tool to use in personalised feedback for those at elevated risk of CVD.

Strengths of the current study include the provision of specific, targeted and individually tailored feedback by a dietitian, made possible through the prior online assessment of dietary intake, provision of current blood lipid results, and an educational counselling session supplemented by a purpose-designed nutrition education manual. Feedback related the individual’s current intakes to appropriate reference ranges and suggested targets in both written and verbal forms, allowing participants time to ask questions and consider results. The use of the Protection Motivation Theory as the intervention framework was appropriate, as participants self-identified as having high serum cholesterol levels (threat vulnerability) and were counselled to achieve self-efficacy through behavioural techniques (Table 1).

Limitations of the current study include the pragmatic study design, relatively small sample size and hence risk of type 2 error. An explanatory study design, such as a randomised controlled trial, may have increased internal validity and given greater proof of causality. However, this study has informed power calculations for future intervention sample sizes, with the primary outcome based on changes in percentage energy of discretionary food choices. The study design was also not truly pragmatic, as an intervention suitable for everyday practice would not have been able to provide shelf-stable grocery products to induce experimentation with unfamiliar products. Given that the greatest dietary change was an approximate 1000 kJ/day reduction in discretionary food choices, experimentation with food choices as a behaviour change may not necessarily be of high value in a brief intervention. Changes in foods recommended for consumption several times a week, such as fish, may not have been accurately captured using the 24-hour recall dietary method, even though both a weekday and weekend day were assessed at each time point. It is possible that this also impacted on legume intake given that they may not be consumed every day [47]. Misreporting is common when assessing dietary intake, although changes in biochemical variables and reduction in BMI in the current study provide support for the relative accuracy of reported dietary intakes [48]. Although the age of the study population reflects those with elevated LDL cholesterol levels within the general population and heart disease currently has the highest mortality rate for women in Australia, this sample was overly represented by females relative to males [49,50]. Moderating contextual factors include the university setting leading to an inherent higher level of trust in the dietary advice, and the majority of participants being recruited from a research volunteer register, hence potentially more likely to comply with recommendations. The number of contacts due to dietary recalls and screening for study entry meant that a relationship was already established between the dietitian and participant, thereby decreasing the time needed for this during the counselling session. The short time-frame is both a strength and a limitation of the current study. The minimum six-week period was sufficient to achieve clinically important changes in plasma lipids, as well as demonstrate to study participants the efficacy of their dietary intervention, based on the short-term dietary changes that they made in that time period. However, these results cannot be generalised into longer-term behaviour changes.

## 5. Conclusions

These findings support the usefulness of including well-structured dietetic counselling by APDs as an initial approach to manage increased risk of CVD conferred by diet. Participants significantly reduced their intakes of energy-dense, nutrient-poor foods, although few significant changes were seen in intakes of specific foods associated with serum lipid reduction. Individuals with hyperlipidaemia can make cumulative small changes to their diet based on the personalised feedback leading to improved CVD risk markers. Recommendations for future practice include utilising validated methods of assessing usual intake prior to counselling sessions in this population and use of behaviour change strategies in tailored interventions for individuals.

## Figures and Tables

**Table 1:** Foods recommended and behaviour change strategies incorporated in the intervention. Foods targeted for dietary knowledge were informed by evidence for reducing cardiovascular disease (CVD) risk [3,28] or improving serum lipids [13].

**Dietary Knowledge**	**Quantity**	**Example Foods**
Nuts	25–30g/day	Unsalted almonds, walnuts
Fish and omega 3 fats	2–3 serves/week	Fresh/canned salmon or tuna
Soy proteins	Up to 7 serves/day	Soy milk, tofu, tempeh
Lentils and legumes	Up to 7 serves/week	Kidney beans, lentils, chick peas
Soluble fibre	Up to 15g/day	Psyllium husk, oat bran, fruit, vegetables
Plant sterols	2–3 serves/day	Margarines/milk/cheese with added sterols
Healthy eating	As given by the Australian Guide to Healthy Eating [31]	
**Behavioural technique [32]**	**Illustration from intervention**	
Provide information about behaviour-health link	General information given about the types of foods and nutrients that increase blood triglyceride levels	
Prompt intention formation	Participants were provided with pantry items of recommended foods and recipes for their use	
Prompt specific goal setting	Participants were required to generate three specific personal goals	
Provide instruction	All participants were provided with 63-page resource manual with recommended serving sizes of food groups and food preparation methods	
Model or demonstrate the behaviour	Study participants were given breakfast upon completion of fasting study measures, with choices offered from a menu of items consistent with the recommended dietary advice	
Provide feedback on performance	Participants were given dietary feedback based on analysis of current intake	

* Other behaviour change techniques such as relapse prevention, time management or prompt barrier identification, may have been incorporated within the individual counselling session dependent on needs identified by Accredited Practising Dietitian.

**Table 2:** Pre- and post-intervention cardiovascular risk factors impacted by dietary intervention. Data reported as mean ± standard deviation, with 95% confidence intervals (CI) for change as given by the coefficient of the regression.

Risk Factors	(Reference Range ^1^)		Baseline (n = 39)	Follow up (n = 38)	Change	95% CI	*p* Value
*Anthropometry*							
BMI		(kg/m^2^)	28.1 ± 5.7	27.6 ± 5.7	−0.4	−0.7, −0.2	<0.01
Weight		(kg)	77.8 ± 17.6	76.3 ± 17.5	−1.3	−1.9, −0.7	<0.01
Waist circumference		(cm)	92.3 ± 15.9	90.2 ± 14.5	−1.5	−2.3, −0.7	<0.01
Blood pressure ^2^		(mmHg)					
*Brachial*							
Systolic			125.9 ± 17.2	121.1 ± 16.0	−4.8	−8.3, −1.2	<0.01
Diastolic			75.9 ± 7.3	73.2 ± 8.6	−2.4	−4.1, −0.7	< 0.01
*Central*							
Systolic			120.2 ± 17.0	116.6 ± 14.7	−3.6	−7.5, 0.2	0.07
Diastolic			77.2 ± 7.3	75.0 ± 8.7	−2.0	−4.0, 0.4	0.06
Augmentation index			108.4 ± 56.8	99.8 ± 34.8	−9.6	−23.1, 3.9	0.16
Fasting Serum CVD risk markers ^3^							
Lipids							
Triglycerides	< 1.5	(mmol/L)	1.60 ± 1.27	1.19 ± 0.51	−0.38	−0.73, −0.03	0.03
Total cholesterol	< 4.00	(mmol/L)	6.79 ± 1.10	6.27 ± 1.00	−0.51	−0.77, −0.24	<0.01
LDL cholesterol	< 2.5	(mmol/L)	4.60 ± 1.04	4.30 ± 0.97	−0.29	−0.52, −0.05	0.02
HDL cholesterol	> 1.0	(mmol/L)	1.46 ± 0.34	1.43 ± 0.35	−0.05	−0.10, −0.00	0.05
Total:HDL ratio			5.02 ± 1.96	4.63 ± 1.44	−0.27	−0.51, −0.04	0.02
Inflammation markers							
*hsCRP*	< 5.0	(mg/L)	2.25 ± 2.26	2.22 ± 2.23	0.1	−0.40, 0.60	0.70
*ALT*	0–45	(U/L)	25.9 ± 15.1	27.8 ± 14.7	2.0	−0.1, 4.1	0.07
*AST*	0–41	(U/L)	25.9 ± 5.9	27.6 ± 7.1	1.9	−0.5, 4.2	0.12
*GGT*	0–45 (F) 0–70 (M)	(U/L)	32.5 ± 37.0	30.1 ± 29.5	−1.1	−5.2, 3.1	0.62
BGL	3.0–6.0	(mmol/L)	5.16 ± 0.57	4.94 ±0.59	−0.20	−0.32, −0.08	<0.01
HOMA IR score ^4^		(mmol/L)	1.04 ± 0.74				

^1^ Reference range (Australian) [34,35]; ^2^ Model includes sex and BMI; ^3^ n = 37 at follow up (n = 1 resumed lipid medication). Models include sex and BMI. PA included in HDL models; ^4^ n = 36 (based on HOMA2 calculations; n = 2 insulin values too low for calculation validity [36]). Abbreviations: BGL, blood glucose level; BMI, body mass index; Coef., Coefficient; F, females; HDL, high density lipoprotein; LDL, low density lipoprotein; M, males; Reference, reference range.

**Table 3:** Pre- and post-intervention dietary intakes of hyperlipidaemic participants as obtained by 24-hour recall. Data presented as mean ± standard deviation and 95% confidence interval for change as given by regression coefficient. Foods specific cardiovascular health also given.

	Baseline (n = 39)	Follow up (n = 38)	Change ^1^	95% CI	*p* Value
Total energy (kJ/day)	9580 ± 2695	8712 ± 2614	−870	−1611, −130	0.02
Discretionary energy (kJ/day) ^2^	3231 ± 2058	2223 ± 1821	−1006	−1563, −450	<0.001
% Discretionary energy	31.7 ± 16.6	24.1 ± 15.1	−7.5	−12.4, −2.7	< 0.01
% Protein	17.4 ± 4.4	18.9 ± 4.6	1.5	0.1, 2.9	0.04
% CHO	45.0 ± 10.0	43.6 ± 9.9	−1.4	−4.4, 1.6	0.35
% Fats	33.6 ± 8.7	32.9 ± 9.7	−0.7	−3.7, 2.3	0.64
% sat. fat	11.4 ± 4.0	9.8 ± 4.5	−1.5	−2.9, −0.2	0.03
% mono. fat	13.5 ± 5.2	12.9 ± 5.2	−0.6	−2.4, 1.2	0.50
% poly. fat	6.0 ± 2.9	7.2 ± 2.8	1.3	0.46, 2.1	<0.01
Fibre (g)	29.2 ± 10.2	29.3 ± 10.0	0.1	−2.2, 2.5	0.91
Sodium (mg)	2764 ± 1397	2410 ± 1184	−358	−650, −67	0.02
Foods specific to cardiovascular health					
Fruit serves/day ^3^	0.85 ± 0.89	0.97 ± 99	0.12	−0.13, 0.37	0.36
Vegetable serves/day ^3^	3.18 ± 2.83	3.05 ± 2.47	−0.13	−0.80, 0.53	0.69
Nuts (g/day)	16.3 ± 32.3	17.6 ± 25.6	1.2	−7.7, 10.2	0.79
Fish (g/day)	29.4 ± 62.9	44.0 ± 68.4	13.5	−5.4, 32.5	0.16
Soy proteins (g/day) ^4^	1.0 ± 2.9	2.9 ± 4.3	2.0	0.8, 3.3	0.001
Legumes (g/day)	8.3 ± 28.0	20.9 ± 54.4	12.6	−0.53, 25.8	0.06
Fibre from oats/psyllium/linseed (g/day) ^5^	1.7 ± 3.0	2.4 ± 3.5	0.6	−0.4, 1.6	0.21
Plant sterols (mg/day)	257 ± 582	604 ± 885	343	74, 611	0.01

^1^ Regression model includes adjustment for recall on weekend/weekday and sex. Coefficient reported for change. Regression model for percentage energy adjusted for recall on weekend/weekday only; ^2^ Energy-dense, nutrient-poor foods classed as discretionary according to the Australian Health Survey discretionary food list [22]; ^3^ Fruit serve = 150g, vegetable serve = 75g; ^4^ Only grams of soy protein reported as this is posited as the factor having lipid-lowering abilities; ^5^ Only grams of fibre reported as this is posited as the mechanism for lowering serum lipids; Abbreviations: PE, percentage energy; CHO, carbohydrates; sat. fats, saturated fats; mono. fats, monounsaturated fats; poly. fats, polyunsaturated fats; g/day, grams per day; mg/day, milligrams per day.

**Table 4:** Reported eating habits of foods related to CVD health.

	Baseline	Follow up	Χ2
	% (n = 39)	% (n = 38) ^1^	*p* Value (Pearsons)
Type of milk normally consumed			
Don’t drink milk	7.7 (n = 3)	7.7 (n = 3)	0.08
Normal/whole/full cream	15.4 (n = 6)	2.6 (n = 1)	
Reduced fat	53.9 (n = 21)	41.0 (n = 16)	
Skim	12.8 (n = 5)	15.4 (n = 6)	
Soy	7.7 (n = 3)	30.8 (n = 12)	
Other/Not sure	2.6 (n = 1)	2.6 (n = 1)	
Type of cheese normally eaten			
Don’t eat cheese	5.1 (n = 2)	5.1 (n = 2)	<0.01
Normal/full fat	56.4 (n = 22)	20.5 (n = 8)	
Reduced fat	28.2 (n = 11)	43.6 (n = 17)	
Low fat	10.3 (n = 4)	30.8 (n = 12)	
Type of meat			
Don’t eat meat	5.1 (n = 2)	5.1 (n = 2)	0.52
Normal/untrimmed	38.5 (n = 15)	23.1 (n = 9)	
Reduced fat/semi-trimmed	35.9 (n = 14)	43.6 (n = 17)	
Low fat/fully-trimmed	20.5 (n = 8)	28.2 (n = 11)	
Type of chicken			
Fried	2.6 (n = 1)	0 (n = 0)	0.38
Crumbed	2.6 (n = 1)	0 (n = 0)	
With skin	20.5 (n = 8)	12.8 (n = 5)	
Skin removed	74.4 (n = 29)	87.2 (n = 34)	
Adding salt to food			
Never add salt	43.6 (n = 17)	51.2 (n = 20)	0.56
During cooking	23.1 (n = 9)	23.1 (n = 9)	
To meals	23.1 (n = 9)	23.1 (n = 9)	
Both meals and cooking	10.3 (n = 4)	2.6 (n = 1)	
Purchasing salt-reduced foods			
Never	18.0 (n = 7)	12.8 (n = 5)	0.12
Sometimes	66.7 (n = 26)	51.3 (n = 20)	
Always	15.4 (n = 6)	35.9 (n = 14)	

^1^ Last observation carried forward for person not completing intervention.

## Supplementary Materials

The following are available online at www.mdpi.com/2227-9032/4/4/75/s1, Figure S1: Study timeline.
