# Supplementary material for: Effectiveness of a Brief Dietetic Intervention for Hyperlipidaemic Adults Using Individually-Tailored Dietary Feedback
Source: Healthcare (Basel). 2016 Oct 11;4(4):75. doi: 10.3390/healthcare4040075 (PMC5198117; doi:10.3390/healthcare4040075)
Supplement: Supplementary file 1 [file healthcare-04-00075-s001.docx]

**Supplementary Materials: Effectiveness of a Brief Dietetic Intervention for Hyperlipidaemic Adults Using Individually-Tailored Dietary Feedback**

Tracy L. Schumacher, Tracy L. Burrows, Megan E. Rollo, Neil J. Spratt, Robin Callister, and Clare E. Collins


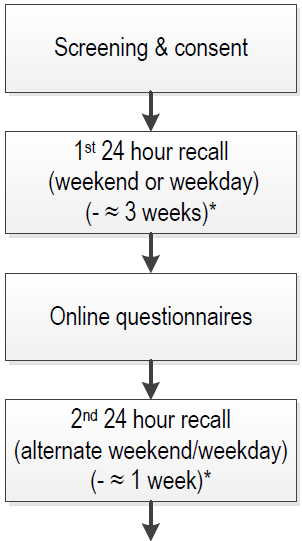


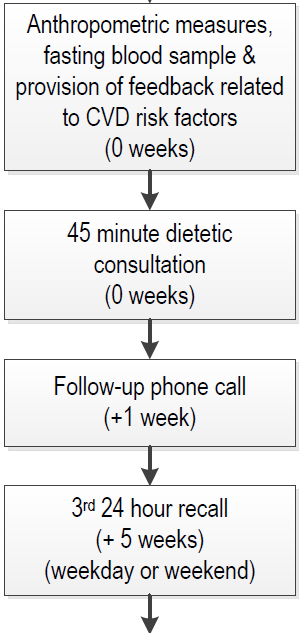


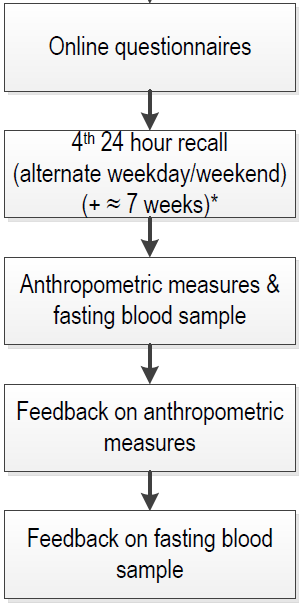


**Figure S1.** Study timeline. * Note that time between recalls and counselling was dependent on completion of online questionnaires, day of original recall and scheduling of appointments.
